# Detection of subclinical myocardial dysfunction in cocaine addicts with feature tracking cardiovascular magnetic resonance

**DOI:** 10.1186/s12968-020-00663-7

**Published:** 2020-09-28

**Authors:** Alicia M. Maceira, Sara Guardiola, Carmen Ripoll, Juan Cosin-Sales, Vicente Belloch, Jose Salazar

**Affiliations:** 1 Cardiovascular Unit, Ascires Biomedical Grup, C/ Marques de San Juan Nº6, 46015, Valencia, Spain; 2 Department of Medicine, Health Sciences School, CEU-Cardenal Herrera University, C/ Santiago Ramón y Cajal, s/n, 46115 Alfara del Patriarca, Moncada-Valencia, Spain; 3 Addictions Treatment Unit of Campanar, La Fe Hospital, Valencia, Spain; 4 Department of Cardiology, Hospital Arnau de Vilanova, Valencia, Spain; 5 Department of Psychiatry, Hospital General Universitario, Valencia, Spain

**Keywords:** Cocaine, Feature tracking, Subclinical dysfunction, Cardiovascular magnetic resonance, Heart failure

## Abstract

**Background:**

Cocaine is an addictive, sympathomimetic drug with potentially lethal effects. We have previously shown with cardiovascular magnetic resonance (CMR) the presence of cardiovascular involvement in a significant percentage of consecutive asymptomatic cocaine addicts. CMR with feature-tracking analysis (CMR-FT) allows for the quantification of myocardial deformation which may detect preclinical involvement. Therefore, we aimed to assess the effects of cocaine on the left ventricular myocardium in a group of asymptomatic cocaine users with CMR-FT.

**Methods:**

In a cohort of asymptomatic cocaine addicts (CA) who had been submitted to CMR at 3 T, we used CMR-FT to measure strain, strain rate and dyssynchrony index in CA with mildly decreased left ventricular ejection fraction (CA-LVEF_d_) and in CA with preserved ejection fraction (CA-LVEF_p_). We also measured these parameters in 30 age-matched healthy subjects.

**Results:**

There were no differences according to age. Significant differences were seen in global longitudinal, radial and circumferential strain, in global longitudinal and radial strain rate and in radial and circumferential dyssynchrony index among the groups, with the lowest values in CA-LVEF_d_ and intermediate values in CA-LVEF_p_. Longitudinal, radial and circumferential strain values were significantly lower in CA-LVEF_p_ with respect to controls.

**Conclusions:**

CA-LVEF_p_ show decreased systolic strain and strain rate values, with intermediate values between healthy controls and CA-LVEF_d_. Signs suggestive of dyssynchrony were also detected. In CA, CMR-FT based strain analysis can detect early subclinical myocardial involvement.

## Background

Cocaine is a highly addictive sympathomimetic drug with potential lethal effects [1] and it is the most commonly used illicit stimulant drug in Europe, where 5.1% of adults are considered to have used cocaine in their lifetime [2]. Cocaine cardiotoxicity appears multifactorial through a variety of mechanisms including myocardial ischemia, [3] systemic hypertension, [4] ventricular hypertrophy, [5] systolic dysfunction, [6] arrhythmias [7] or myocarditis [8]. These deleterious effects may be increased with the concomitant use of other substances, mainly etanol and tobacco [9].

We have previously shown, [10] using a comprehensive cardiovascular magnetic resonance (CMR) protocol at 3 T, cardiovascular involvement in 71% of subjects in a reasonably large cohort of consecutive, non-selected, asymptomatic chronic cocaine addicts (CA). Cardiovascular involvement consisted mainly of mild left ventricular (LV) dilatation, dysfunction and hypertrophy, right ventricular (RV) dysfunction and focal LV myocardial fibrosis.

Global volumetric measures of myocardial function may not be sensitive enough to identify early myocardial dysfunction. The measurement of myocardial deformation by strain analysis is an evolving tool to quantify regional and global myocardial function [11]. It may detect subclinical dysfunction and provide prognostic information in several pathologies, [12–17] with strain/strain rate measurements becoming increasingly common both in clinical practice and research studies.

CMR with feature tracking analysis (CMR-FT) allows for a fast, less observer-dependent assessment of strain. CMR-FT provides tracking of tissue voxel motion of cine-CMR images with a potential to assess longitudinal, circumferential and radial myocardial strain, strain rate, displacement and torsion, independent of additional sequences and with a good agreement versus myocardial tagging with harmonic phase imaging as a reference standard, [18] but with reduced post processing time and with no additional sequences needed, allowing for CMR-FT to be applicable both in prospective and retrospective studies.

There is increasing evidence that a reduction of LV strain and strain rate precedes the haemodynamic effects of LV impairment. This has been shown mainly with speckle tracking echocardiography (STE) in anthracycline induced cardiotoxicity, where the reduction in strain parameters precedes the decrease of ejection fraction, [12, 19] and in some cardiomyopathies [20, 21]. Therefore, we aimed to quantify global LV strain and strain rate in our cohort of asymptomatic cocaine users, in order to assess whether this approach could detect subclinical systolic dysfunction.

## Methods

### Subject recruitment and CMR protocol

The protocol and subject recruitment have already been published elsewhere [10]. Briefly, this was a prospective study carried out with CMR at 3 T for which we recruited consecutive asymptomatic CA) (18–60 years) who fulfilled the criteria for cocaine addiction or abuse, [22] who were attending a rehabilitation clinic and had been off-cocaine for a maximum of 3 months. We have now evaluated with CMR-FT these subjects, classified according to the presence of mildly decreased LV ejection fraction (CA-LVEF_d_), defined as LVEF below the 95% CI of the reference for the subject’s age and gender, according to our own reported reference values, [23] or preserved LVEF (CA-LVEF_p_). We also included 30 age-matched healthy subjects scanned at 3 T. The study was approved by the institutional ethics committee. All the subjects included were informed about this study and permission was requested to further analyze their CMR studies. No patient was excluded due to poor images and all subjects could be analysed with FT-CMR.

The study was done on a 3 T CMR scanner (Achieva 3 T TX, Philips Healthcare, Best, The Netherlands). The comprehensive acquisition protocol and sequence parameters employed have already been reported [10]. Of note, high resolution balanced steady state free precession (bSSFP) end-expiratory breath-hold cines were acquired using retrospective electrocardiogram (ECG) triggering with subsequent contiguous short-axis cines from the atrioventricular ring to the apex and acquisition of 2-chamber, 4-chamber and 3-chamber view cines. Typically, 40 phases were acquired in each cine sequence with an average temporal resolution of 21 ± 1.5 ms. As part of the initial protocol, dipyridamole (0.84 mg/Kg in 6 min) stress and rest myocardial perfusion images were acquired in the first 48 subjects by using a saturation prepared gradient-echo sequence in three ventricular short-axis sections during gadolinium bolus administration (0.1 mmol/Kg). In the subsequent 46 subjects the same myocardial perfusion sequence was acquired only at rest. Late gadolinium enhancement (LGE) sequences were acquired after gadolinium bolus administration in all subjects.

### Feature tracking analysis

A dedicated CMR-FT software package (CVI42, version 5.10.1, Circle Cardiovascular Imaging Inc., Calgary, Canada,) that allows for the measurement of two-dimensional strain derived parameters based on user definition of myocardial borders on standard cine bSSFP images was used.

For the short axis stack analysis, all the short axis slices showing the endocardial cavity surrounded by myocardium through all the phases along the cardiac cycle were included, thus avoiding the distortion due to the LV outflow tract in the very basal slices. For long axis analysis, 2 and 4-chamber cines were used.

Endocardial and epicardial borders were manually drawn at end-diastole in all short and long axis cines, excluding papillary muscles from the endocardial contour. An automated tracking algorithm was applied in all the cine sequences throughout the cardiac cycle. Endocardial contours were manually drawn in all analyzed slices by an experienced observer (SG, 2-year experience in CMR-FT) who was blind to the group of pertenance of the subjects included. Typical intra and interstudy reproducibility of CMR-FT derived strain/strain rate measurements in our unit as well as our analysis methodology have been previously reported [24].

Tracking performance was visually reviewed in all the slices to ensure accurate tracking. Tracing of the myocardial borders was manually adjusted in case of inadequate automated border tracking. Long-axis cines were tracked to derive longitudinal strain parameters. From the short axis stack three planes were identified and used to derive circumferential and radial strain parameters at the basal, midventricular and apical level, carefully assuring that all three planes exhibited circular myocardium around the LV cavity throughout the cardiac cycle. Peak global systolic radial strain (GRS), circumferential strain (GCS) and longitudinal (GLS) strains were quantified. Global peak systolic radial (GRSR), circumferential (GCSR) and longitudinal (GLSR) strain rates were also obtained from the peak of the averaged curve. LV longitudinal, circumferential and radial systolic dyssynchrony index (L-SDI, C-SDI, R-SDI) were calculated as the standard deviation (SD) of the calculated time to peak strain percentages of the cardiac cycle with segmental strain analysis [25].

### Statistical analysis

Continuous variables were found to satisfy a normal distribution using the Shapiro-Wilks test and are presented as mean ± SD. Categorical data were presented as percentages. Variables regarding baseline characteristics, ventricular dimensions and function and myocardial deformation parameters were compared using ANOVA test for continuous variables, including alcohol abuse and presence of LGE and LV hypertrophyas covariates, with Tukey’s HSD for post-hoc test. Chi-square was used for categorical and qualitative variables. All statistical analyses were done using SPSS statistical software version (v17.0, Statistical Package for the Social Sciences, International Business Machines, Inc., Armonk, New York, USA).

## Results

Of our initial cohort of 94 CA studied, we have used CMR-FT to analyse LV and RV strain and strain rate, and LV dyssynchrony, in 62 subjects with preserved ejection fraction (CA-LVEF_p_) and in 32 subjects with mildly decreased ejection fraction (CA-LVEF_d_). A third group of healthy non-CA controls, age and gender matched, scanned at 3 T was included as a reference. This control group included 30 healthy subjects, all of which were asymptomatic, with normal ECG, no family or personal history of cardiopathy, free of cardiovascular disease risk factors and found to have a normal CMR.

### Baseline characteristics, anthropometric variables

The main anthropometric variables along with data about cardiovascular disease risk factors in the three groups are presented in Table 1. The majority of subjects included were males, with no differences in gender distribution or age among the groups. No significant differences were found in height, weight, heart rate and blood pressure among the groups. As expected, the most prevalent risk factor among CA was current smoking. The presence of symptoms such as chest pain, shortness of breath, palpitations or syncope had been extensively investigated and none of the subjects included referred any symptoms.

**Table 1:** Biological data, laboratory findings and cardiovascular risk factors

Group	Controls	CA-LVEF_p_	CA-LVEF_d_	P
N	30	62	32	
Age (yrs)	37 ± 10	35 ± 7	38 ± 7	0.167
Gender (% males)	93%	84%	90%	0.095
Heart rate (bpm)	71 ± 14	67 ± 11	69 ± 11	0.550
SBP (mmHg)	130 ± 12	127 ± 11	129 ± 11	0.228
DBP (mmHg)	76 ± 10	83 ± 11	84 ± 11	0.167
Weight (kg)	82 ± 13	78 ± 13	84 ± 16	0.181
Height (cm)	170 ± 7	172 ± 7	175 ± 5	0.066
Body mass index (kg/m2)	27 ± 3	26 ± 4	28 ± 7	0.059
Dyslipidaemia, n (%)	0 (0%)	6 (9.6%)	4 (12.5%)	< 0.001
Smoking habits				
Currently smoking, n (%)	0 (0%)	47 (76%)	24 (75%)	< 0.001
Ex–smoker, n (%)	0 (0%)	3 (4.8%)	2 (6.2%)	0.417
Years of smoking habitus	–	14 ± 9	13 ± 10	
Cigarettes per day	–	13 ± 12	15 ± 13	
Hypertension, n (%)	0 (0%)	2 (3.2%)	2 (6.2%)	0.397

*CA-LVEF*_*p*_ cocaine addicts with preserved left ventricular systolic function, *CA-LVEF*_*d*_ cocaine addicts with mildly decreased left ventricular systolic function, *SBP* systolic blood pressure, *DBP* diastolic blood pressure

Data on cocaine and alcohol use in the two CA groups are shown in Table 2. No significant differences were seen between both groups for any of the parameters investigated, including duration of addiction and amount of consumption. Noteworthy, the time interval between the last episode of cocaine uptake and CMR was similar, 72 ± 63 days for CA-LVEF_p_ and 73 ± 79 days for CA-LVEF_d_. Regarding alcohol consumption, both groups of CA were homogeneous with regard to prevalence, amount and frequency of consumption as well as years of alcohol abuse duration. When alcohol consumption was included as covariate in the statistical analysis we did not find a significant effect of this variable on any of the analyzed deformation parameters.

**Table 2:** Data on cocaine and alcohol use

	CA-LVEF_p_	CA-LVEF_d_	P
Cocaine			
Age at the time of first use, yrs	22.8 ± 6.9	22.0 ± 6.9	0.594
Time between last use and CMR (days)	72 ± 63	73 ± 79	0.959
Frequency of use in the last month of consumption (number of uses)	3.1 ± 1.7	3.4 ± 1.8	0.351
Frequency of use in the last 3 months of consumption (uses per month)	3.7 ± 1.6	3.9 ± 1.4	0.215
Maximum frequency of use in lifetime (uses per month)	7.3 ± 1.5	7.5 ± 1.5	0.511
Amount of consumption in the last month of consumption (g)	3.7 ± 1.7	4.1 ± 1.3	0.280
Years of regular cocaine use (yrs)	13.8 ± 13	14.2 ± 8	0.793
Route of administration			
Nasal insufflation	82%	81%	
Smoked	15%	15%	
Intravenous	3%	4%	0.922
Alcohol			
Subjects with alcohol abuse/dependence, n (%)	32 (52%)	17 (53%)	0.663
Years of alcohol use	9.7 ± 11.2	10.6 ± 11.5	0.712
Amount of consumption in the past month (g)	101 ± 138	73 ± 142	0.415

*CA-LVEF*_*p*_ cocaine addicts with preserved systolic function, *CA-LVEF*_*d*_ cocaine addicts with mildly decreased systolic function

### CMR findings

**Fig. 1:**
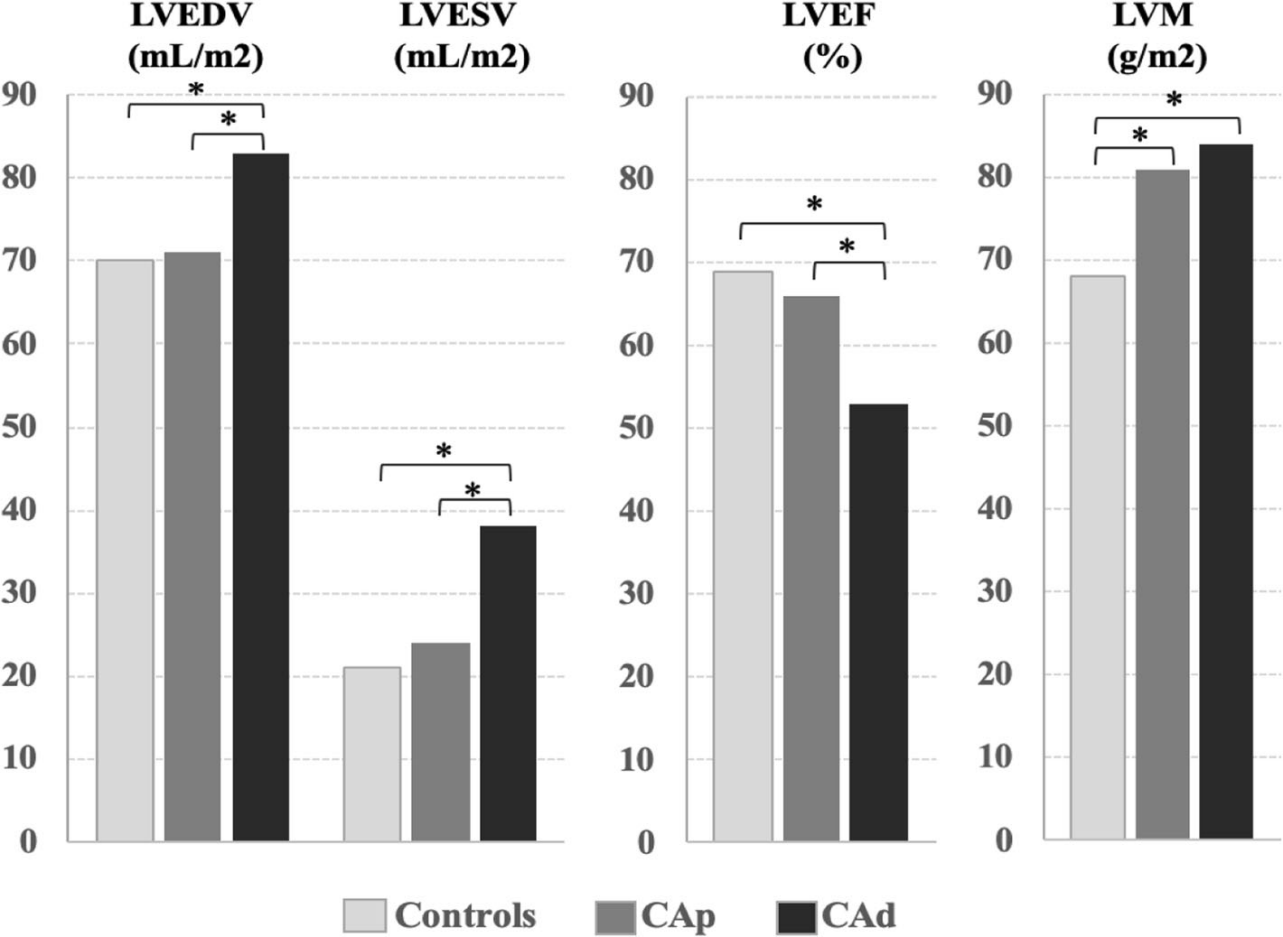
Differences in cardiovascular magnetic resonance (CMR) derived left ventricular (LV) parameters among the three groups. LVEDV, left ventricular end-diastolic function (mL/m2), LVESV, left ventricular end-systolic function (mL/m2), LVEF, left ventricular ejection fraction (%), LVM, left ventricular mass (g/m2), CA_p_, cocaine addicts with preserved systolic function, CA_d_, cocaine addicts with decreased systolic function. *, *p* < 0.05 on post-hoc analysis

CMR derived cardiac dimensions and function from the three groups are compared in Table 3. As expected, there were significant differences for LV and RV volumes and LV mass among the three groups. In the post-hoc analysis (Fig. 1), the only LV parameters that showed differences between controls and CA-LVEF_p_ were LV mass (LVM) index and relative wall mass (RWM) that was calculated as the ratio between mass and end-diastolic volume, while no RV parameter showed differences between controls and CA-LVEF_p_. The majority of LV and RV parameters showed differences between CA-LVEF_p_ and CA-LVEF_d_.

**Table 3:** CMR derived parameters in the three groups

	Healthy Controls	CA-LVEF_p_	CA-LVEF_d_	P
LVEDV (mL/m2)	70 ± 13	72 ± 14	83 ± 12	0.001
LVESV (mL/m2)	21 ± 6	26 ± 6	38 ± 6	< 0.001
LVEF (%)	69 ± 6	65 ± 5	54 ± 3	< 0.001
LVM (g/m2)	68 ± 10	79 ± 13	84 ± 12	< 0.001
RWM (g/mL)	0.99 ± 0.15	1.10 ± 0.23	1.04 ± 0.19	0.003
LVH (n, % of subjects with LVH)	0, 0	17, 28	11, 34	0.001
LGE (n, % of subjects with LGE)	0, 0	18, 30	10, 33	0.001
LGE (gr)	0	0.79 ± 0.18	0.89 ± 0.23	0.089
LGE (% of LV myocardium)	0	0.54 ± 0.13	0.55 ± 0.13	0.265
RVEDV (mL/m2)	73 ± 13	79 ± 15	87 ± 12	0.004
RVESV (mL/m2)	28 ± 7	34 ± 9	41 ± 7	< 0.001
RVEF (%)	62 ± 6	58 ± 5	52 ± 4	< 0.001

*LV* left ventricle, *EDV* end-diastolic volume, *ESV* end-systolic volume, *EF* ejection fraction, *LVM* left ventricular mass, *RWM* relative wall mass, *LVH* left ventricular hypertrophy, *RV* right ventricle

There were no differences in the prevalence of LVH, defined as LVM index above the 95% CI of the reference for the subject’s age and gender [23], between both groups of CA (*P* = 0.629). LVH was present in 29% of CA-LVEF_p_ and 31% of CA-LVEF_d_, but it was mild in all cases, with maximum LVM of 108 g/m2 in CA-LVEF_p_, and maximum LVM of 113 g/m2 in CA-LVEF_d_. Differences were seen in LV volumes between controls and CA-LVEF_d_, and between CA-LVEF_p_ and CA-LVEF_d_. 1.6% of CA-LVEF_p_ had LV dilatation, with maximum LVEDV of 101 mL, while 19% of CA-LVEF_d_ had LV dilatation, with maximum LVEDV of 106 mL/m2. No subject had LVEDV below the inferior limit of normal.

Finally, there were no differences in the prevalence of LGE between both CA groups. LGE was detected in 18 subjects in the CA-LVEF_p_ group (10 intramyocardial, 7 in the inferior ventricular junction and 1 subepicardial) and in 10 individuals in the CA-LVEF_d_ group (4 intramyocardial, 1 subendocardial, 1 subepicardial and 4 in the inferior ventricular junction) (*P* = 0.811). Importantly, LGE was of very limited extension in all the subjects LGE+. This was quantified for CA-LVEF_p_ in 0.79 ± 0.18 g, 0.54 ± 0.13% of the LV myocardium, and for CA-LVEF_d_ in 0.89 ± 0.23 g, 0.55 ± 0.13% of the LV myocardium (*P* = 0.099 and 0.417 respectively).

### CMR-FT derived parameters

Noteworthy, we have previously analyzed and reported the intra- and inter-observer variability of CMR-FT measurements in our unit [24]. More recently, with the same CVI42 software version has been as used in this study, our typical intraclass correlation coefficient (ICC) for LV strain and strain rate analysys was typically above 0.95 for intraobserver reproducibility and above 0.86 for interobserver reproducibility We have also analysed our reproducibility for RV strain and strain rate analysis which produced ICC above 0.87 for intraobserver reproducibility and above 0.77 for interobserver reproducibility, (unpublished observation). Data on CMR-FT derived parameters are shown in Table 4.

**Table 4:** CMR-FT derived parameters

	Healthy Controls	CA-LVEF_p_	CA-LVEF_d_	P
LV_GLS (%)	−18.24 ± 2.7	−14.1 ± 2.4	−12.4 ± 3.7	< 0.001
LV_GRS (%)	33.5 ± 11.3	26.4 ± 9.2	19.8 ± 9.5	0.001
LV_GCS (%)	− 18.5 ± 5.3	− 15.0 ± 3.8	− 11.7 ± 4.7	< 0.001
LV_L-SDI (%)	8.95 ± 3.5	10.4 ± 5.8	14.9 ± 8.0	0.019
LV_R-SDI (%)	7.1 ± 2.8	10.0 ± 4.7	13.9 ± 8.1	0.001
LV_C-SDI (%)	8.0 ± 3.4	9.7 ± 4.7	14.8 ± 9.1	0.002
LV_GLSR (s^− 1^)	− 111 ± 22	−92 ± 28	−81 ± 25	0.003
LV_GRSR (s^−1^)	228 ± 99	188 ± 104	142 ± 70	0.042
LV_GCSR (s^− 1^)	− 125 ± 32	− 118 ± 100	− 80 ± 62	0.110
RV_GLS (%)	− 19.3 ± 2.7	− 16.3 ± 3.3	− 15.1 ± 2.7	0.002
RV_GRS (%)	40.9 ± 10.9	29.8 ± 9.0	27.0 ± 7.5	0.003
RV_GLSR (s-1)	− 129 ± 34	− 109 ± 30	− 78 ± 63	0.025
RV_GRSR (s-1)	241 ± 61	181 ± 63	167 ± 39	0.031

*LV* left ventrticle, *RV* right ventricle, *CA-LVEF*_*p*_ cocaine addicts with preserved systolic function, *CA-LVEF*_*d*_ cocaine addicts with mildly decreased systolic function, *GLS* global longitudinal strain, *GRS* global radial strain, *GCS* global circumferential strain, *GLSR* global longitudinal strain rate, *GRSR* global radial strain rate, *GCSR* global circumferential strain rate, *L-SDI* longitudinal systolic dyssynchrony index, *R-SDI* radial systolic dyssynchrony index, *C-SDI* circumferential systolic dyssynchrony index

### Strain

**Fig. 2:**
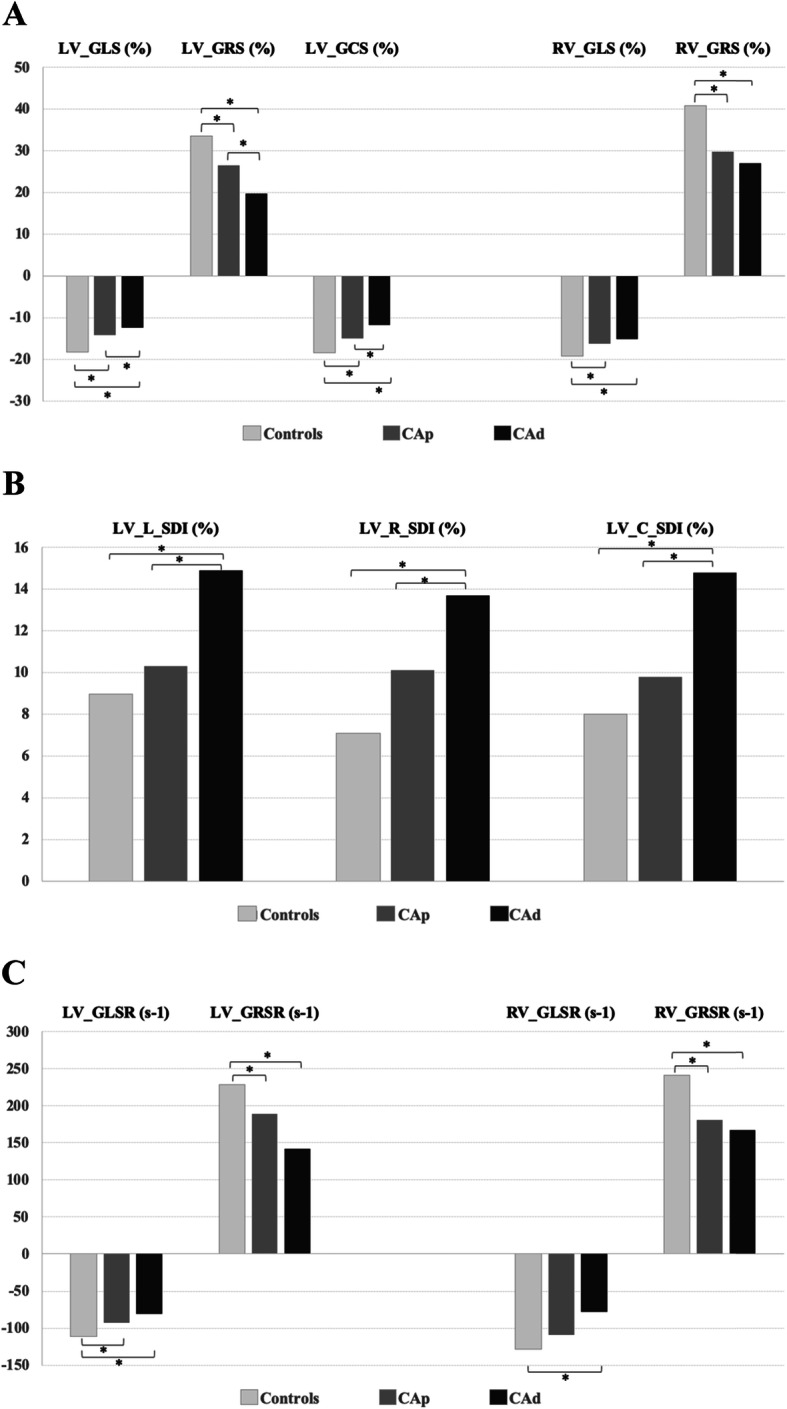
Differences in CMR feature tracking (CMR-FT) derived myocardial deformation parameters among the three groups. GLS, global longitudinal strain (%); GRS, global radial strain (%); GCS, global circumferential strain (%); GLSR, global longitudinal strain rate (s-1); GRSR, global radial strain rate (s-1); L-SDI, longitudinal systolic dyssynchrony index (%); R-SDI, radial systolic dyssynchrony index (%); C-SDI, circumferential systolic dyssynchrony index (%), CA_p_, cocaine addicts with preserved systolic function, CA_d_, cocaine addicts with decreased systolic function. *, *p* < 0.05 on post-hoc analysis

In a per-group analysis, significant differences were seen in LV GLS, GRS, and GCS among the three groups with all of them being significantly decreased in CA-LVEF_d_, as expected. As shown in Table 4, the CA-LVEF_p_ group showed for all types of strain intermediate values between controls and CA-LVEF_d_, with significant differences among the three groups for all types of strain. In the post-hoc analysis (Fig. 2a), both LV GLS, GRS and GCS were significantly lower in CA-LVEF_p_ compared to healthy controls, while CA-LVEF_d_ exhibited the lowest values with significant differences with respect to both CA-LVEF_p_ and controls.

We also analysed RV global longitudinal and radial strain, significant differences were seen in RV GRS and GLS among the three groups, as depicted in Table 4. Both of them were lowest in CA-LVEE_d_, as expected. In the post-hoc analysis both RV GRS and GLS were significantly decreased in CA-LVEF_p_ and CA-LVEF_d_ compared to healthy controls, while no significant differences were seen between CA-LVEF_p_ and CA-LVEF_d_.

### Dyssynchrony index

As shown in Table 4, significant differences were found for LV L-SDI, C-SDI and R-SDI among the groups, with all of them being higher in CA-LVEF_d_. CA-LVEF_p_ showed intermediate values between healthy controls and CA-LVEF_d_. Post-hoc analysis (Fig. 2b) showed that differences reached statistical significance between CA-LVEF_d_ and healthy controls for all three variables. Equally, L-SDI, C-SDI and R-SDI were also significantly different between CA-LVEF_d_ and CA-LVEF_p_. No significant differences were seen between controls and CA-LVEF_p_ for either of these parameters.

### Strain rate

Longitudinal, radial and circumferential systolic strain rate parameters were measured in all subjects. Significant differences were seen for LV GLSR, GRSR) (Table 4) with both being lowest in CA-LVEF_d_, as expected, while CA-LVEF_p_ exhibited intermediate values in all parameters. The post-hoc analysis (Fig. 2c) showed that both parameters were significantly lower in CA-LVEF_d_ compared to healthy controls, and in CA-LVEF_p_ compared to controls, while no significant differences were seen between CA-LVEF_p_ and CA-LVEF_d_.

We also analysed RV GLS and GRS, significant differences were seen for both parameters among the three groups (Table 4). Both of them were lowest in CA-LVEF_d_. The post-hoc analysis showed that RV GRSR was significantly decreased in CA-LVEF_p_ and CA-LVEF_d_ compared to controls, with no significant differences seen between CA-LVEF_p_ and CA-LVEF_d_, and RV GLSR was only significantly decreased in CA-LVEF_d_ compared to healthy controls.

### LV hypertrophy, stress perfusion, LGE and deformation parameters

**Fig. 3:**
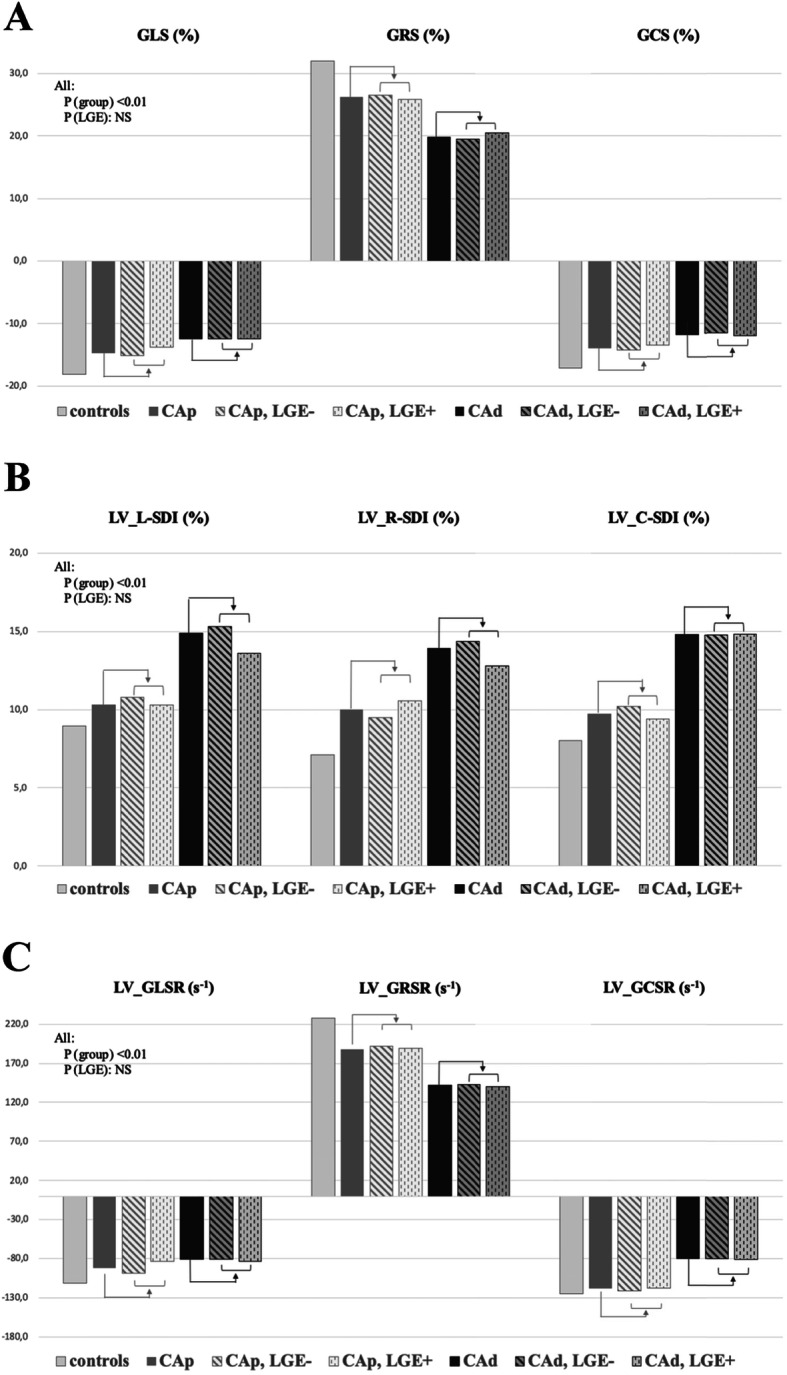
Graphical representation of subgroup distribution with regard to the presence of late gadolinium enhancement (LGE). GLS, global longitudinal strain (%); GRS, global radial strain (%); GCS, global circumferential strain (%); GLSR, global longitudinal strain rate (s-1); GRSR, global radial strain rate (s-1); L-SDI, longitudinal systolic dyssynchrony index (%); R-SDI, radial systolic dyssynchrony index (%); C-SDI, circumferential systolic dyssynchrony index (%), CA_p_, cocaine addicts with preserved systolic function, CA_d_, cocaine addicts with decreased systolic function. *, *p* < 0.05 on post-hoc analysis

LV hypertrophy (LVH) was present in roughly one third of subjects in the CA-LVEF_p_ group and in the CA-LVEF_d_ group, and in all cases was mild, as depicted above. LVH was included as covariate in the statistical analysis of all deformation parameters and was found to have no significant effect of this variable on these parameters. LV dilatation was also seen marginally (1 subject) in CA-LVEFp and no subject exhibited LV volumes below the inferior limit of the normal range. Two subjects in the CA-LVEF_p_ group and two in the CA-LVEF_d_ group had shown mild (1 segment), short-lasting subendocardial perfusion abnormalities on stress perfusion, but we did not find a significant effect of this covariate on strain parameters. Finally, LGE was of very limited extension in all the LGE+ subjects, as presented above. Still, LGE was also included as covariate in the statistical analysis of all deformation parameters. We did not find a significant effect of this variable on any of the analyzed parameters. Figure 3 shows in graphical form the subgroup distribution with regard to presence of LGE.

## Discussion

As far as we are aware, this is the first report on CMR-FT at 3 T to study differences in myocardial deformation in CA with preserved and with mildly decreased LV systolic function in comparison with a group of healthy controls as a reference. We obtained several findings. Firstly, CMR-FT LV and RV deformation analysis showed significant and progressive decrease in GLS, GRS and GCS from healthy controls to CA-LVEF_d_ with all of them significantly decreased in CA-LVEF_p_ compared to healthy controls. Secondly, LV L-SDI, C-SDI and R-SDI, as well as LV and RV GLSR and LV GRSR showed significant differences among the groups, but only LV GLSR, GRSR and RV GRSR showed differences between healthy controls and CA-LVEF_p_, while neither dyssynchrony nor other strain rate parameters were significantly different in CA-LVEF_p_ with respect to controls. Lastly, we also found that LVM and RWM were significantly increased in CA-LVEF_p_ with respect to healthy controls.

### Analysis of myocardial deformation

There is increasing evidence that a reduction of LV strain and strain rate precedes the hemodynamic effects of LV impairment [26, 27] and may add prognostic information in a number of conditions [28]. Thus, we hypothesized that deformation analysis measured with CMR could detect preclinical involvement in CA with still preserved LVEF. Although CMR tagging is the gold-standard technique for deformation analysis, [29–32] it is time-consuming and generally limited to the research arena requiring specific imaging protocols, sequences and dedicated post-processing software. CMR-FT is being increasingly used for deformation analysis since it does not suffer from the same limitations as echocardiography-based techniques while it can be done off-line in previously acquired standard cine sequences [33] while enjoying short processing times [34]. Equally, CMR-FT of the RV offers the potential for rapid and sensitive quantification of function.

### Strain analysis

Strain is the most frequently used deformation index. Usually, 2D CMR-FT is used, as in this study, although 3D CMR-FT has been reported to be more reproducible [35]. Still, the latter relies on the measurements done in the 2D short axis cine stack, so it is still affected by the through-plane motion of the heart. In our study CA-LVEF_p_ exhibited for all types of strain intermediate values between controls and CA-LVEF_d_, and post-hoc analysis showed that LV GLS, GRS, GCS, and RV GRS and GLS were already decreased in CA-LVEF_p_. Regarding LV strain parameters, there are no previous reports with which we could compare our results, but similar findings have been reported with STE in patients presenting with anthracycline induced cardiotoxicity, in whom a decrease of GLS [12] and GCS [36, 37] have been reported to precede significant changes in LVEF, and in alcohol induced cardiomyopathy, in which both GLS and GCS are reduced in subclinical myocardial dysfunction [38]. Noteworthy, in our study GRS was also able to detect subclinical dysfunction despite the higher reported measurement variability of GRS, [24, 39–41] which usually requires greater sample sizes than other strain parameters in order to detect differences. Regarding RV strain parameters, again there are no previous reports for comparison, but it has been shown with STE that RV strain analysis is able to detect early affection of RV function in patients under chemotherapy, which in a particular study was associated with reduced recovery of LVEF [42].

We do not know the exact mechanism by which a decrease in global strain occurs in CA-LVEF_p_. Actually, cocaine chronic cardiotoxicity has been considered to be a multifactorial process plus individual susceptibility, and it can be expected that this is the case in CA. Decreased LV volume has been reported to explain reduced GLS or GCS with preserved EF, [43] but in our study no subject had LV volume below the normal range. We included in our analysis the presence of LGE, LVH and alcohol consumption as potential covariates. Interestingly, we observed decreased GLS along with increased LV mass in CA-LVEF_p_, which is in accordance with previous observations of decreased longitudinal function in hypertrophied ventricles, [44] but LVH failed to show a significant effect on strain parameters in the statistical analysis. With respect to presence of hypertension and alcohol consumption, both groups of CA had similar number of hypertensives and alcohol abusers, and similar severity of alcohol abuse, and in fact these variables failed to show a significant effect as covariates on strain parameters. Limited, short-lasting, subendocardial stress myocardial perfusion defects had been observed in 4 of the first 48 subjects of the cohort in which stress perfusion study was done, but this was not a covariate with significant effect on any strain parameter. Regarding LGE, this was of limited extension in our subjects and, though we observed a progressive decline in strain from the control group down to CA-LVEF_d_ with LGE, the presence of LGE was not a covariate with significant effect in any strain parameter. Again we cannot compare our results with those of others but it has been reported, in conditions such as hypertrophic cardiomyopathy, [21] that strain and strain rate values are further decreased in those patients who exhibit LGE. We think that the very limited amount of LGE detected in our study can explain our findings. Interestingly, interstitial fibrosis might be present in the myocardium of CA which could affect strain measurements. Although we could not use the T1 mapping technique, we hypothesize that it would provide interesting data with regard to the myocardial interstitium in cocaine cardiotoxicity. Furthermore, T1 mapping along with strain analysis might provide a more comprehensive and accurate evaluation of this condition. We also assessed whether increased LV volumes occurred in CA-LVEF_p_, since strain measures are still slightly affected by LV preload, [45] but we found no significant LV dilatation in CA-LVEF_p_ compared with controls.

Finally, we speculate that the presence of reduced strain values in CA-LVEF_p_ might have independent prognostic importance, similarly to other conditions, [46] though our study was not designed to assess outcomes. Future studies will show the prognostic value of abnormal strain measurements and whether they normalize after cocaine cessation.

### Dyssynchrony index

Segmental strain analysis with CMR-FT is a useful method for assessment of regional myocardial deformation [35]. We had previously observed in our cohort of CA a variable degree of dyssynchrony of contraction on visual assessment. Therefore, in this study we intended to assess left ventricular dyssynchrony with CMR-FT by measuring SDI, defined as the standard deviation of the calculated time-to-peak percentages of all segments with respect to the duration of the cardiac cycle [25]. CMR-FT derived SDI has demonstrated good agreement with dyssynchrony measured with STE [47] and has been used in conditions such as heart failure and congenital heart disease [25, 48]. Also, LV dyssynchrony may be a sign of impaired LV function in CA similarly to other conditions [25].

Though we observed significant differences among the groups for L-SDI, R-SDI and C-SDI, with the healthy control group showing the lowest values and CA-LVEF_d_ exhibiting the highest, neither of these parameters could exhibit significant differences between controls and CA-LVEF_p_. We obtained a C-SDI of 8.0 ± 3.4% in the control group, higher than previously published ranges, [25] though differences in design and methodology as well as the sample size, could explain the differences. We cannot compare our findings in CA with other reports, but equivalent results have been shown in alcohol induced cardiomyopathy with realtime 3D echocardiography, with increased SDI in addicts compared to controls [49].

### Strain rate

Significant differences were found for LV and RV GLSR and GRSR among the groups, and post-hoc analysis showed for both LV GLSR and LV GRSR, and for RV GRSR, significant differences between controls and CA-LVEF_p_, and between controls and CA-LVEF_d_. Usually, in CMR-FT analysis integral variables such as displacement and strain are more reliable than the differential ones, including strain rate. In particular, temporal resolution is such that rapid myocardial events like isovolumic time intervals are not reliable. Strain rate measurements are noisier and less reproducible than strain parameters, and more affected by temporal resolution, thus results must be interpreted with caution. Actually, CMR-FT has lower temporal resolution compared to STE, which explains why CMR-FT strain values tend to be lower [50] and strain rate parameters less robust. As for strain and dyssynchrony, neither alcohol consumption nor the presence of LVH or LGE were found to have a significant effect on these parameters.

### Ventricular dimensions and function

We observed significant differences in all LV and RV dimensions and function parameters mainly due to differences between controls and CA-LVEF_d_. LVH was present in nearly one third of both groups of CA but it was in mild all cases, though both LV mass and relative wall mass, which is calculated as mass divided by the end-diastolic volume, were significantly increased in CA-LVEF_p_ compared to controls. Equally, LV dilatation was seen marginally in CA-LVEF_p_ and in 19% of CA-LVEF_d_, but again was mild in all cases. Thus, LV mass and dimensions were just mildly affected in our cohort of asymptomatic CA, and LVH was not found to have a significant statistical effect on deformation parameters. We hypothesize that several factors not fully understood, might interact causing the decreased strain we have found in these subjects.

### Clinical implications of deformation analysis in CA

Regarding the clinical use of CMR-FT in cocaine cardiotoxicity, we consider that this technique cannot be promoted in CA before several questions are answered, as was the case with other pathologies such as chemotherapy induced cardiotoxicity. First, the prognostic significance of strain measurement in cocaine addicts should be established. Also, decision on which single parameter to use, and with which cut-off values, should be made. Finally, strong evidence should be available before any changes in medical management can be discussed. At the moment we can only suggest to use strain parameters, because of their known robustness, and among them global longitudinal strain would be our measurement of choice, similar to other pathologies. Still, data on prognostic value is mandatory before we can promote this technique in clinical practice and, at current, no data is available in order to change this subject’s medical management further than strongly recommend cessation of cocaine and a closer follow-up, with otherwise treatment according to current guidelines.

## Limitations

Several limitations should be considered. Regarding the CA subjects included, notwithstanding the difficulty in recruiting consecutive asymptomatic cocaine users, there is a potential selection bias since they were referred from the addictions treatment unit, which might indicate a more severe addiction or higher suspicion of heart disease. Also, although our sample size is one of the largest published to date on cocaine toxicity studied with CMR, it remains fairly limited in power to detect small differences among the three groups, especially for less robust parameters such as strain rate. CA are usually dependent of several drugs and in real life it is extremely difficult to recruit single-drug addicts. Therefore we could not refuse subjects with alcohol abuse for the sake of a timely recruiting which, even then, took years. Still, the two groups of CA were homogeneous with regard to number of alcohol abusers, amount and frequency of consumption and years of alcohol abuse duration.

Regarding the technique used, we only analyzed global strain and strain rate parameters, since these have been shown to be more robust [10, 35]. Regional abnormalities could then potentially go undetected if compensated by other regions. CMR-FT has not yet been validated against the clinical gold standard sonomicrometry. Still, it has been validated against artificially-prepared phantom images, [51] and against CMR tagging [18, 52, 53] with reasonable agreement, mainly for GLS and GCS. CMR-FT seems to be technique, acquisition and vendor dependent [45, 54] and pooled means, though valuable and a reasonable guide for CMR-FT users, [55] may not be directly transferrable to a particular patient group. For this reason we did not carry out a per patient analysis. It is essential to develop reference standard for each technique and analytical product for clinical use, and to sequentially compare patient data using the same software.

## Conclusions

The results of our study indicate that CMR-FT based myocardial deformation analysis with measurement of myocardial strain could allow for a reliable detection of LV early involvement in CA with preserved LVEF.

## Data Availability

All data generated or analysed during this study are included in this published article.

## Abbreviations

bSSFP: Balanced steady state free precession
C-SDI: Circumferential systolic dyssynchrony index
CA: Cocaine addicts
CA-LVEF_p_: Cocaine addict with preserved systolic function
CA-LVEF_d_: Cocaine addict with decreased systolic function
CMR: Cardiovascular magnetic resonance
CMR-FT: CMR with feature-tracking
ECG: Electrocardiogram
GCS: Global circumferential strain
GCSR: Global circumferential strain rate
GLS: Global longitudinal strain
GLSR: Global longitudinal strain rate
GRS: Global radial strain
GRSR: Global radial strain rate
L-SDI: Longitudinal systolic dyssynchrony index
LGE: Late gadolinium enhancement
LV: Left ventricle/left ventricular
LVEDV: Left ventricular end-diastolic volume
LVEF: Left ventricular ejection fraction
LVESV: Left ventricular end-systolic volume
LVH: Left ventricular hypertrophy
LVM: Left ventricular mass
R-SDI: Radial systolic dyssynchrony index
RV: Right ventricle/right ventricular
RWM: Relative wall mass
STE: Speckle tracking echocardiography
